# Radiomic-Based Machine Learning Classifiers for HPV Status Prediction in Oropharyngeal Cancer: A Systematic Review and Meta-Analysis †

**DOI:** 10.3390/diagnostics16010068

**Published:** 2025-12-24

**Authors:** Anna Luíza Damaceno Araújo, Luiz Paulo Kowalski, Alan Roger Santos-Silva, Brendo Vinícius Rodrigues Louredo, Cristina Saldivia-Siracusa, Otávio Augusto A. M. de Melo, Deivid Cabral, Andrés Coca-Pelaz, Orlando Guntinas-Lichius, Remco de Bree, Pawel Golusinski, Karthik N. Rao, Robert P. Takes, Nabil F. Saba, Alfio Ferlito

**Affiliations:** 1 Head and Neck Surgery Department and LIM 28, University of São Paulo Medical School, São Paulo 05508-020, Brazil; luiz.kowalski@hc.fm.usp.br; 2 Hospital Israelita Albert Einstein, São Paulo 05652-900, Brazil; 3 Department of Head and Neck Surgery and Otorhinolaryngology, A.C. Camargo Cancer Center, São Paulo 01509-010, Brazil; 4 Faculdade de Odontologia de Piracicaba, Universidade Estadual de Campinas (FOP-UNICAMP), Piracicaba 13414-903, Brazil; 5 Department of Otolaryngology, Hospital Universitario Central de Asturias, University of Oviedo, ISPA, IUOPA, CIBERONC, 33011 Oviedo, Spain; acocapelaz@yahoo.es; 6 Department of Otorhinolaryngology, Jena University Hospital, 07740 Jena, Germany; 7 Department of Head and Neck Surgical Oncology, University Medical Center Utrecht, 3508 GA Utrecht, The Netherlands; r.debree@umcutrecht.nl; 8 Department of Otolaryngology and Maxillofacial Surgery, University of Zielona Gora, 65-046 Zielona Gora, Poland; 9 Department of Maxillofacial Surgery, Poznan University of Medical Sciences, 61-701 Poznan, Poland; 10 Department of Head and Neck Oncology, Sri Shankara Cancer Foundation, Bangalore 560004, India; karthik.nag.rao@gmail.com; 11 Department of Otorhinolaryngology and Head and Neck Surgery, Radboud University Medical Center, 6525 GA Nijmegen, The Netherlands; robert.takes@radboudumc.nl; 12 Department of Hematology and Medical Oncology, Emory University, Atlanta, GA 30322, USA; nfsaba@emory.edu; 13 International Head and Neck Scientific Group, 35100 Padua, Italy; profalfioferlito@gmail.com

**Keywords:** HPV, imaging, machine learning, oropharyngeal cancer, radiomics

## Abstract

**Background:** The aim of the present systematic review (SR) is to compile evidence regarding the use of radiomic-based machine learning (ML) models for predicting human papillomavirus (HPV) status in oropharyngeal squamous cell carcinoma (OPSCC) patients and to assess their reliability, methodological frameworks, and clinical applicability. The SR was conducted following PRISMA 2020 guidelines and registered in PROSPERO (CRD42025640065). **Methods**: Using the PICOS framework, the review question was defined as follows: “Can radiomic-based ML models accurately predict HPV status in OPSCC?” Electronic databases (Cochrane, Embase, IEEE Xplore, BVS, PubMed, Scopus, Web of Science) and gray literature (arXiv, Google Scholar and ProQuest) were searched. Retrospective cohort studies assessing radiomics for HPV prediction were included. Risk of bias (RoB) was evaluated using Prediction model Risk Of Bias ASsessment Tool (PROBAST), and data were synthesized based on imaging modality, architecture type/learning modalities, and the presence of external validation. Meta-analysis was performed for externally validated models using MetaBayesDTA and RStudio. **Results**: Twenty-four studies including 8627 patients were analyzed. Imaging modalities included computed tomography (CT), magnetic resonance imaging (MRI), contrast-enhanced computed tomography (CE-CT), and ^18^F-fluorodeoxyglucose positron emission tomography (^18^F-FDG PET). Logistic regression, random forest, eXtreme Gradient Boosting (XGBoost), and convolutional neural networks (CNNs) were commonly used. Most datasets were imbalanced with a predominance of HPV+ cases. Only eight studies reported external validation results. AUROC values ranged between 0.59 and 0.87 in the internal validation and between 0.48 and 0.91 in the external validation results. RoB was high in most studies, mainly due to reliance on p16-only HPV testing, insufficient events, or inadequate handling of class imbalance. Deep Learning (DL) models achieved moderate performance with considerable heterogeneity (sensitivity: 0.61; specificity: 0.65). In contrast, traditional models provided higher, more consistent performance (sensitivity: 0.72; specificity: 0.77). **Conclusions**: Radiomic-based ML models show potential for HPV status prediction in OPSCC, but methodological heterogeneity and a high RoB limit current clinical applicability.

## 1. Introduction

The association between high-risk human papillomavirus (HPV) infection and oropharyngeal squamous cell carcinomas (OPSCC) has been consistently demonstrated. Recognition of HPV-positive OPSCC as a distinct pathological entity has led to its classification with unique staging rules in the UICC/AJCC 8th edition Tumor–Node–Metastasis (TNM) system, reflecting its significantly more favorable prognosis compared to HPV-negative carcinomas [1,2]. Affected patients are usually younger, healthier, non-smoking individuals, with mortality reported to be significantly lower [3]. Given the superior survival outcomes, significant clinical research efforts are now focused on de-intensifying therapy (e.g., reduced radiation dose, transoral surgery alone) for HPV-positive OPSCC patients. The primary goal of these de-escalation trials is to maintain high cure rates while minimizing treatment-related morbidity. Consequently, accurate and accessible determination of HPV status is not merely a diagnostic exercise but a critical step towards delivering personalized care [4,5,6].

It is well established that HPV status should be confirmed using at least two complementary methods (e.g., p16 immunohistochemistry combined with HPV-specific testing), as relying solely on HPV DNA genotyping or p16 expression may lead to misclassification and inflated prevalence estimates. However, these methods may not be readily available in all clinical settings, such as smaller community hospitals, resource-limited laboratories, or centers without specialized molecular pathology facilities [7]. In these cases, p16 immunohistochemistry may be sufficient as a standalone test for HPV infection status in tumors with appropriate morphology [8]. In contrast, medical imaging modalities such as sonography, computed tomography (CT), magnetic resonance imaging (MRI), and positron emission tomography CT (PET/CT) are routinely used for staging and treatment planning. This accessibility has driven the growth of radiomics, which involves the high-throughput extraction of quantitative features from medical images to characterize tumor shape, texture, and intensity patterns. Machine learning (ML) algorithms can then analyze these data to uncover complex relationships and build predictive models for diagnosis and prognosis. Radiomics aims to link the underlying tumor biology with its radiographic phenotype. Integrating radiomic features with modern ML techniques, including advanced artificial intelligence (AI) models such as convolutional neural networks (CNNs), has the potential to strengthen the role of imaging biomarkers in translational research, improving patient stratification and risk assessment [9,10].

However, the path to clinical application faces challenges. There is no consensus regarding the impact of equipment and image acquisition techniques on the stability of radiomic features [11]. Furthermore, while some prior studies have shown limitations in segmentation strategies and a lack of robust results [12,13,14,15], more recent studies have demonstrated great performance metrics, with area under the receiver operating characteristic curve (AUROCs) reaching over 90% in some cases [11,16,17]. A reliable radiomic biomarker could therefore have significant clinical utility, enabling retrospective HPV analyses when tissue samples are unavailable, assisting in cases of cancers of unknown primary (CUP), or serving as a screening tool for at-risk individuals in regions without routine HPV testing [18]. More importantly, by providing a potentially standardized and accessible means of stratification, radiomics could support the broader clinical adoption of personalized treatment protocols (i.e., de-intensification, standard or intensified therapy) [4,5,6]. Additionally, HPV testing on cytology specimens from cervical lymph node metastasis, when combined with radiomic analysis, may enhance the accuracy of HPV status determination and support more precise patient stratification [19].

The present systematic review (SR) aims to retrieve and summarize good-quality evidence regarding the use of radiomic signatures extracted from medical images for HPV status prediction in OPSCC patients. Specifically, this review aims to evaluate the diagnostic performance of these radiomic-based ML models and to analyze the methodological frameworks adopted across different imaging modalities, with the goal of assessing their current feasibility and reliability for clinical deployment. Therefore, this SR and meta-analysis aims to assess the diagnostic accuracy and methodological robustness of radiomic-based ML models for HPV status prediction in OPSCC.

## 2. Methods

### 2.1. Protocol and Registration

The present SR was conducted in accordance with the Preferred Reporting Items for Systematic Reviews and Meta-Analyses (PRISMA) 2020 guidelines [20,21] and the PRISMA-P checklist [22,23]. Given that this review focuses on diagnostic test accuracy, it also adhered to the PRISMA-DTA statement [24] to ensure transparent and standardized reporting of diagnostic accuracy meta-analyses. The protocol was prospectively registered in the International Prospective Register of Systematic Reviews (PROSPERO) under the registration number CRD42025640065. The review protocol defined inclusion and exclusion criteria, data extraction forms, and pre-specified subgroup analyses to ensure methodological transparency (Table S1).

### 2.2. Eligibility Criteria

The PICOS framework yielded the following focused review question: “Can radiomic-based ML models accurately predict HPV status in OPSCC?” We defined the Population, Intervention, Comparator, Outcome, and Study design (PICOS) a priori, and no deviations from the original protocol occurred during the review process. Population was patients diagnosed with OPSCC; Interventions were radiomic-based ML classifiers; Comparators were not applicable in this systematic review; Outcomes were the performance of radiomic-based models for HPV status prediction; and the included Studies were retrospective cohort studies. Studies combining the HPV status with other relevant descriptors to predict tumor volume, cancer progression, risk stratification, locoregional recurrence, survival prediction, treatment individualization or response were not included.

### 2.3. Information Sources and Search Strategy

Individualized search strategies were conducted on 7 November 2024 for the following electronic databases: Cochrane Library, Embase, IEEE Xplore, Portal Regional da Biblioteca Virtual em Saúde (BVS), PubMed, Scopus, and Web of Science. Gray literature was included to minimize publication bias and to identify potentially unpublished protocols. The gray literature search encompassed the following sources: arXiv, Google Scholar, and ProQuest. The reference list of included articles and other SRs were manually screened to identify any records that were not retrieved through database search. The complete search strategy is shown in Table A1 and Table A2 in Appendix A.

### 2.4. Selection Process

Initially, duplicate records were automatically removed using Endnote [25] and Rayyan [26]. An additional manual exclusion step was necessary to address the records retrieved from IEEE Xplore, arXiv, and the Cochrane Library that could not be exported in RIS format. Once duplicates were removed, two reviewers (A.L.D.A. and C.S.S.) independently conducted the first phase of study selection by reading titles and abstracts. The eligibility criteria were applied only to those articles that remained after the first phase (sought for retrieval). Divergences were reviewed by a third reviewer (L.P.K.).

### 2.5. Data Collection Process and Data Items

Two reviewers (A.L.D.A. and L.P.K.) independently conducted the screening of articles by reading the titles and abstracts and excluding those that clearly did not fulfill the eligibility criteria. In the second phase, the two reviewers read the full texts of the papers to identify the eligible articles. Discrepancies were resolved by consensus.

Rayyan QCRI [26] was used as the reference manager to perform the screening of articles, exclude duplicates, and record the primary reasons for exclusion. Data extraction was conducted by the primary researcher (A.L.D.A.) and guided by a tailored data extraction form originally suggested by The Cochrane Collaboration. Qualitative and quantitative data were tabulated and processed in Microsoft Excel^®^. The main data extracted included the following: author, year, source of volume of interest (VOI), total images/patients per subset and per HPV status, HPV status test, strategies to mitigate data imbalance, imaging modality, radiomic features, feature selection method, classifier, AUROC with confidence interval, standard deviation, and standard error, true positive (TP), false negative (FN), true negative (TN), and false positive (FP) values, explainability methods, study limitations, and conclusions.

### 2.6. Risk of Bias (RoB) Assessment

Each study was assessed independently by two authors (A.L.D.A. and L.P.K.) through the Prediction model Risk of Bias Assessment Tool (PROBAST) for assessing the RoB and applicability of diagnostic and prognostic prediction model studies [27,28].

### 2.7. Effect Measures

The effect measures included the area under the receiver operating characteristic curve (AUROC) and the corresponding confidence intervals, which are not inherently affected by class imbalance and allow for the assessment of class separability and the generalization ability of the models. For meta-analyses, TP, FN, TN, and FP values are required.

### 2.8. Synthesis Methods

The synthesis of data was performed based on imaging modality, model architecture/learning type, and the presence of external validation. The meta-analysis was conducted by selecting the best performance models reported in studies conducting external validation. Since the studies are diverse in methods, we grouped the models into three main groups: (1) Deep Learning (DL) models, (2) Traditional models with their respective feature selection methods, and (3) Ensemble and Gradient Boosting methods with associated feature selection methods. The meta-analysis was conducted using The MetaBayesDTA (v1.5.2) [29], an extension of the MetaDTA web application [30,31,32], a Shiny application developed by the group at the Centre for Reviews and Dissemination (CRD), University of York, designed to perform diagnostic accuracy meta-analysis (particularly involving sensitivity and specificity).

Between-study heterogeneity was planned to be evaluated both visually and statistically. Initially, heterogeneity was to be explored through the inspection of summary receiver operating characteristic (sROC) plots and confidence regions generated by the MetaBayesDTA package, allowing for qualitative assessment of variability across studies. In addition, quantitative measures of heterogeneity, including Cochran’s Q statistic, τ^2^ (tau-squared), and I^2^, were calculated using RStudio (version 2025.09.2-418). These metrics were used to estimate the magnitude and proportion of total variability attributable to differences between studies rather than to sampling error.

## 3. Results

### 3.1. Study Selection

**Figure 1:**
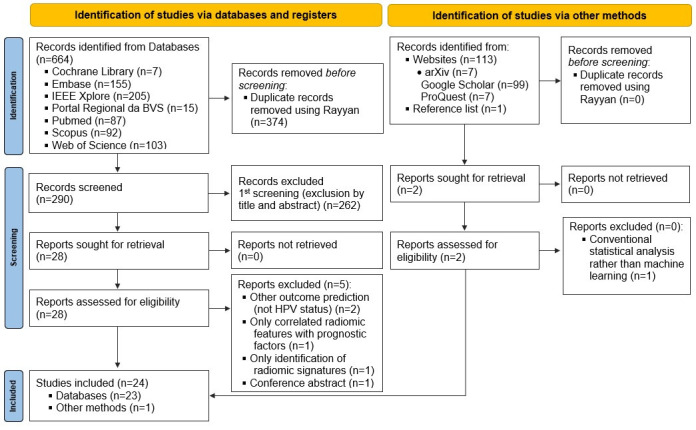
PRISMA Flowchart. This diagram illustrates the identification, screening, eligibility assessment, and inclusion of studies evaluating radiomics and machine learning approaches for HPV status prediction. A total of 24 studies were included in the final qualitative synthesis. BVS: Biblioteca Virtual em Saúde; IIEE: Instituto dos Engenheiros Eletrônicos e Eletricistas.

Amongst a total of 777 records identified through the search strategy, twenty-four articles [11,16,17,18,33,34,35,36,37,38,39,40,41,42,43,44,45,46,47,48,49,50,51,52] fulfilled the eligibility criteria and were included in this SR. The study selection process is summarized in the PRISMA Flowchart (Figure 1) and the reasons for exclusion of each article read in full text in the second phase are described in Table A3 in Appendix B.

### 3.2. Study Characteristics

A total of 8627 patients with OPSCC were included for the development and validation of HPV status detection models, with some risk of patient duplication since studies from the same research groups were included in this SR. Concerning dataset size, four studies included fewer than 100 patients [18,48,51,52]; twelve included between 100 and 300 patients [11,16,33,34,36,37,38,42,43,46,47,50]; four included between 300 and 500 patients [17,39,40,41]; two included between 500 and 1000 patients [44,45]; and two included more than 1000 patients [34,49].

Confirmatory HPV-specific testing in addition to p16 immunohistochemistry, using at least two combined methods as recommended by American Society of Clinical Oncology (ASCO) guidelines [7], was conducted in six studies. The specific HPV tests included polymerase chain reaction (PCR) [36,52], DNA in situ hybridization (DNA-ISH) [44], and VirusSeq [42], with some studies applying more than one specific method [40,41]. Seventeen studies relied solely on p16 immunohistochemistry (IHC p16) as a surrogate marker, and two studies [33,48] did not report the HPV testing method used.

Methodologically, studies were vastly diverse with main variations residing in source of VOI and imaging modalities. The imaging modalities were predominantly computed tomography (CT [17,18,34,35,39,40,42,44,48,49], followed MRI [16,36,37,38,43,47,50,51,52], contrast-enhanced computed tomography (CE-CT) [11,33,45], and ^18^F-fluorodeoxyglucose positron emission tomography (^18^F-FDG PET) [41,43,46]. Two studies explored multiple imaging modalities [40,43]. Only three studies made clear that images were obtained prior to the treatment [11,51,52]. The majority of studies were developed based on gross primary tumor volume (GTVp) [33,34,35,36,37,38,39,41,42,43,44,45,47,49,50,51], five were based on GTVp and gross nodal tumor volume (GTVn) [11,16,17,46,52], one applied GTVp, GTVn and parotid [18], one used the cubic region of interest (ROI) of the oropharynx, not precise anatomical delineation [48], and one compared GTVp, GTVn, GTVp + GTVn, and consensus of all lymph nodes [40]. When comparing approaches according to the VOI, best results were achieved when associating primary tumor and nodal VOIs [48]. Three studies [36,37,42] also developed clinical and multimodal models (i.e., radiomic + clinical data) for comparison. Most studies’ samples were highly imbalanced [11,17,33,36,39,40,41,43,44,45,46,47,49,50,51,52], with a predominance of HPV+ patients [11,16,17,33,37,39,40,43,44,45,46,47,48,49,50,51,52] and only four with more HPV- patients [18,34,36,38]. Four studies were slightly imbalanced [16,34,37,48], one was moderately imbalanced [18], and only one was balanced [38]. Three studies did not report the proportion of HPV+ patients in the cohort used [35,41,42]. Some studies applied strategies to mitigate data imbalance as synthetic minority over-sampling technique (SMOTE) [43,47,51], t-distributed Stochastic Neighbor Embedding (t-SNE) [49], Random Over-Sampling Examples (ROSE) [33], oversampling [50], or class-weighted approach [40]. Only eight studies [11,16,39,40,41,42,44,50] reported external validation results. Regarding the performance, AUROC values ranged between 0.59 and 0.87 in the internal validation and between 0.48 and 0.91 in the external validation results. Feature selection methods varied a lot with recursive feature elimination (RFE) [16,36,37,38,47], linear regression with the least absolute shrinkage and selection operator (LASSO) [36,45,51,52] and hierarchical clustering [35,40] being the most common. With respect to HPV status prediction classifiers, Logistic regression (LR) was the most commonly used model [34,35,36,37,38,42,45,46,47,48,51,52] followed by random forest (RF) [18,36,48,49,50,52], and eXtreme Gradient Boosting (XGBoost) [18,40,41,52]. Two studies explored multilayer perceptron (a feed-forward neural network architecture that learns nonlinear associations between high-dimensional image-derived features and clinical outcomes such as HPV status) and fully connected networks [18,50]. Only four studies employed convolutional neural network approaches, including Inception V3 [39], ElNet [40], 3D and 2D CNN architectures [44], and ResNet-18 [48]. Explainability methods included saliency maps [42], Shapley Additive exPlanations (SHAP) plots [36,43], and Gradient-weighted Class Activation Mapping (grad-CAM) [39]. In addition to the main goal, which was HPV status prediction, one study assessed the impact of artifacts on model performance [33] and one study evaluated the influence of CT scanner manufacturer on the robustness of radiomic features [49]. The studies’ characteristics are summarized in Table 1.

### 3.3. RoB in Studies

Retrospective cohorts are the most common study design used for prediction model development. Prior to model training, exclusions may occur but are typically well justified (e.g., poor-quality MRI, missing HPV status). Therefore, in the participant domain, all studies were rated as having a low RoB and low concern regarding applicability.

For the predictor domain, an unclear RoB was noted when it was not explicitly stated whether the predictor assessors (particularly those extracting radiomic features) were blinded to HPV status. A high risk was assigned when predictors used in model development were not available prior to treatment, compromising the model’s intended applicability (i.e., pre-treatment use). However, all included studies developed their models using pre-treatment variables (clinical assessment and imaging).

For the outcome domain, an unclear or high RoB was assigned in the absence of information regarding whether pathologists were blinded to predictor data, and when HPV status was determined solely based on p16 immunohistochemistry. The exclusive use of p16 as a surrogate marker is not considered adequate according to current clinical guidelines (e.g., ASCO), which recommend dual testing for accurate HPV status determination [7].

For the analysis domain, an unclear or high RoB was assigned when one or more of the following conditions were present: an insufficient number of events (HPV-positive cases); predictor selection based solely on univariable analyses (e.g., univariable Cox or logistic regression, *t*-tests, Kolmogorov–Smirnov tests, or biserial correlation); inadequate handling of class imbalance; the absence of modeling for center effects despite the use of multi-institutional data; no mention of missing data or imputation strategies; failure to account for stratification, clustering, or other forms of data dependency; and a lack of appropriate methods to mitigate overfitting or assess model optimism, such as nested cross-validation with Bayesian optimization, bootstrapping, or penalization techniques. Additionally, if the dataset was imbalanced, authors should have applied strategies to mitigate this imbalance; if no such strategies were conducted, we considered this a source of bias. In this systematic review, we prioritized the AUROC, which was reported in all included studies. However, even in the absence of AUROC, studies that reported other relevant performance metrics such as accuracy, sensitivity, specificity, precision, and/or F1-score were considered to have a low RoB.

A high RoB was seen in twenty-one studies [11,16,17,18,33,34,35,36,37,38,39,42,43,44,45,46,47,48,49,50,51] and an unclear RoB in three studies [40,41,52] (Table 2). No applicability concerns were raised.

### 3.4. Meta-Analysis

To streamline the subgroup analysis for our meta-analysis, we consolidated external validation results. From each paper, we selected only the best-performing model from each main methodological category (i.e., DL models, Traditional models, and Ensemble/Gradient Boosting methods) for inclusion. However, a significant limitation emerged, as most studies did not report the confusion matrix (TP, FN, FP, TN) required for the meta-analysis, nor did they report the performance metrics needed to calculate them. In the absence of reported confusion matrices, we derived them from the reported cohort sizes and available performance metrics (e.g., sensitivity and specificity). These derived matrices were cross-verified by recalculating the published performance results. The reconstructed values showed minor inconsistencies compared with the reported outcomes. We proceeded with the planned meta-analysis, assuming that these discrepancies were likely attributable to rounding errors, as the resulting distributions of TP, FN, FP, and TN appeared plausible given the sample sizes of the external validation cohorts. Nevertheless, since these derived data may introduce estimation bias, the pooled results should be interpreted with caution, and this limitation was considered when assessing the robustness of the meta-analytic estimates.

**Figure 2:**
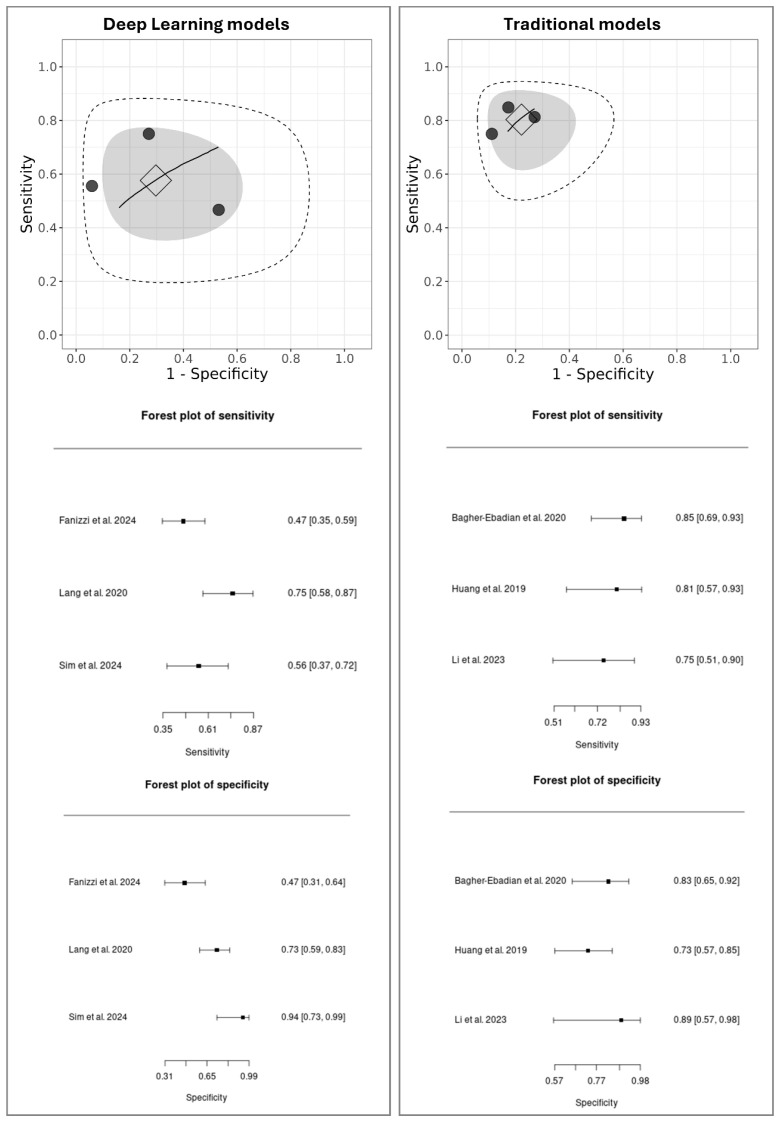
MetaBayesDTA framework. Summary of Receiver Operating Characteristic (sROC) curves and forest plots of sensitivity and specificity for DL models [39,44,50] versus Traditional models [11,16,42]. Individual studies are represented as black dots, with their dispersion reflecting heterogeneity. The square indicates the summary point (pooled sensitivity and specificity), estimated using a bivariate model. The inner gray ellipse represents the 95% confidence region around the summary point, while the outer dashed ellipse shows the 95% prediction region, indicating where results of future studies would be expected to fall. A wider prediction region suggests greater heterogeneity. The sROC curve represents the overall trade-off between sensitivity and specificity across different diagnostic thresholds, with proximity to the top-left corner indicating better diagnostic performance.

Three studies were selected for the DL meta-analysis [39,44,50] and three for the Traditional model’s meta-analysis [11,16,42]. For the Ensemble and Gradient Boosting Methods, only one study [50] provided external validation results; therefore, a meta-analysis could not be conducted for this group. The results of the meta-analysis are presented in Figure 2, which shows the sROC curves for both DL and Traditional models. Aiming for transparency, we also provide a summary table (Table 3) showing the TP, FN, FP, and TN values used in the meta-analysis.

In the sROC plots, each black dot represents an individual study, with their spread indicating the heterogeneity between studies. The square marks the pooled diagnostic performance, the gray shaded ellipse of its 95% confidence region, and the larger dashed ellipse of the 95% prediction region, which reflects the expected range for future studies.

A direct comparison reveals a notable performance difference. The DL models showed moderate pooled performance (sensitivity: 0.61; 95% CI: 0.35–0.87, specificity: 0.65; 95% CI: 0.31–0.99) with considerable heterogeneity, as indicated by the wide dispersion of individual studies and a large prediction region. In contrast, the Traditional models demonstrated higher, more consistent performance (sensitivity: 0.72; 95% CI: 0.51–0.93, specificity: 0.77; 95% CI: 0.57–0.98), with low variability among studies and a tighter prediction ellipse, suggesting good consistency and stable performance across the included studies.

To further quantify heterogeneity among the included studies, an additional analysis was performed using RStudio (version 2025.09.2-418). The pooled estimates indicated substantial heterogeneity, with a Q statistic of 29.47 (df = 5, *p* < 0.001), τ^2^ = 1.66, and I^2^ = 78.2%, suggesting considerable between-study variability. The pooled sensitivity was 0.855 (95% CI = 0.763–0.915) and the pooled specificity was 0.799 (95% CI = 0.654–0.893). These findings confirm the presence of notable heterogeneity, consistent with the variability observed in the sROC plots generated by MetaBayesDTA.

## 4. Discussion

The present SR aimed to evaluate and synthesize evidence on the diagnostic performance of ML models for HPV status prediction in OPSCC. To our knowledge, this is the first systematic review with meta-analysis focusing exclusively on external validation results comparing Traditional and DL approaches. Similar reviews as the one by Chen et al. [53] focused on parameters obtained from structural and diffusion-weighted MRI. Although their methodological approach differed substantially from the present SR, their findings align with ours, indicating that in ML studies, predictive performance significantly improves when clinical variables are incorporated into the models, compared with models relying solely on imaging features. Song et al. [54] reported a pooled sensitivity and specificity of 79% and 75%, respectively, and performed subgroup analyses stratified by imaging modality, validation type, sample size, and geographic region. Most recently, Ansari et al. [55] conducted a well-structured systematic review with meta-analysis that reported a pooled sensitivity of 77.2% and specificity of 76%. Their review encompassed studies largely overlapping with those included in our analysis, concluding that the diagnostic accuracy of radiomics-derived features from medical imaging remains inferior to that achieved by established para-clinical IHC techniques. In their meta-analysis, the authors stratified the sROC curves according to imaging modality, distinguishing between MRI- and CT-derived datasets. In contrast, our work offers a complementary perspective by concentrating on the performance of externally validated models, regardless of the imaging source. We believe this focus provides an additional and valuable dimension to the existing evidence. Both types of meta-analytical aggregation (i.e., by imaging modality and by validation strategy) are informative and should be jointly considered by researchers in this field.

To date, no clear recommendations exist regarding the reliability and clinical applicability of these models, due to methodological heterogeneity and limited external validation. In Section 4, we will address specific aspects aimed at highlighting the most pertinent questions regarding the optimal methodologies and common findings of the included studies, particularly focusing on which features are more suitable for model training, whether it is preferable to use only images or to combine them with clinical data, which data augmentation strategies are most used, and which explainability methods were explored. Considerations about the performance and RoB will also be discussed.

### 4.1. Radiomic Features Related to Tumor Characteristics

Radiomic features are known to reflect the biological differences between HPV-positive and HPV-negative tumors. However, the exploration of such features is specific to each imaging modality and depends on the completeness and quality of the dataset. In this section, we summarize the most discriminative features reported across studies to support methodological choices in future research.

In the context of MRI, according to Boot et al. [36], the features *compactness1*, *compactness2* and *sphericity* were more discriminative of HPV-positive tumors, which tend to be more spherical, while the *major and minor axis length* are more discriminative of HPV-negative tumors. Additionally, the features *coefficient of variation* and *quartile coefficient* were also pointed to be correlated with higher intensity heterogeneity in HPV-negative tumors. Suh et al. [52] indicated that *lower mean and median Apparent Diffusion Coefficient* (ADC) histograms were indicative of more homogeneous tumor tissue, along with higher *kurtosis* and *skewness*. In the model developed by Jo et al. [43], which integrated radiomic features from MRI and 18F-FDG PET/CT, *SUVmax* was identified as the most relevant variable.

For studies applying CE-CT, Altinok et al. [33] identified *Sphericity* and *Max2D-DiameterRow* as the key radiomic features in their Bayesian Network model. The authors noted that higher values of *Max2D-DiameterRow* reflect greater complexity in tumor morphology. These features are considered particularly informative, as tumors with well-defined borders and a more rounded shape are characteristic of HPV-positive cases. Similarly, Leijenaar et al. [45] reported that the radiomic features highlighted by their predictive models suggest that HPV-positive tumors tend to exhibit greater uniformity and homogeneity, reduced contrast enhancement, lower minimum density values, and increased variability in intensity across neighboring voxels. These imaging characteristics are associated with *Gray-level size zone matrix*, *small zone emphasis*, *Gray-level co-occurrence matrix inverse variance* and *Laplacian of Gaussian (4 mm) 10th percentile*, which may correspond to underlying histopathological traits of HPV-positive tumors, such as lobular growth patterns and lymphocytic infiltration; however, confirming these associations requires further investigation using datasets enriched with histological information. In addition, *Gray-level size zone matrix*, *low gray-level large size emphasis*, and *Laplacian of Gaussian (3 mm) kurtosis* have been linked to HPV-positive tumors that demonstrate lower contrast uptake and more extreme density values [11].

In studies using CT imaging, Prasse et al. [18] reported that two features were repeatedly selected in the best-performing models: *original_firstorder_Minimum* in the GTV and *original_firstorder_RootMeanSquared* in the parotid. These findings support the hypothesis that HPV infection may leave detectable signs even in non-tumoral tissues, although the relevance of the parotid as a predictive VOI remains inconclusive regarding its causal role. Similarly, Yu et al. [17] observed that HPV-positive patients tended to have smaller and geometrically simpler tumors, highlighting tumor size and shape as important features. Following this approach, the features most indicative of HPV status included *MeanBreadth*, associated with tumor size, with HPV-positive patients typically exhibiting smaller tumors, and *SphericalDisproportion*, reflecting tumor morphology and suggesting a simpler, less irregular shape in HPV-positive cases. Consistent with these observations, Bogowicz et al. [34] found that tumor heterogeneity was the key feature influencing model performance, emphasizing the importance of intratumoral variability in characterizing HPV-related tumors.

### 4.2. Explainable AI (XAI)

The correlation of such features with clinically interpretable characteristics requires extensive investigation, as radiomic parameters often capture abstract image patterns that are not directly visible to the human eye. In this context, the integration of explainable artificial intelligence (XAI) approaches represents a significant advancement, as they can help uncover the biological or morphological meaning behind radiomic signatures. However, only a few studies to date have incorporated explicit explainability methods, and their application remains largely limited to post hoc visualizations or feature importance analyses. Future research should therefore prioritize the use of interpretable models to strengthen the clinical reliability and translational potential of radiomics-based AI systems.

Saliency maps as reported by Lv et al. [46] highlight which regions of an image most strongly influence the model’s decision. In practice, they assign an “importance value” to each pixel. SHAP plots [36,43] are used mainly with tabular or numerical data (like radiomic features). They show how much each feature contributes to the model’s final prediction, which helps identify which radiomic features are most relevant for distinguishing between classes (e.g., HPV-positive vs. HPV-negative tumors). The grad-CAM visualization as reported by Fanizzi et al. [39] is exclusive for CNNs. It uses the gradients of the output layer to create a heatmap over the original image, highlighting the regions that most influenced the model’s classification. This allows researchers to check whether the model is focusing on meaningful anatomical or pathological areas. The t-SNE reported by [49] is a dimensionality reduction technique that projects high-dimensional data into two or three dimensions, allowing visually similar points to cluster together and revealing patterns or subgroups within the data. These methods may introduce optimism if not adequately assessed.

Given the growing complexity of AI models in radiomics, we recommend the routine incorporation of explainability methods in future studies. Using XAI approaches not only improves transparency and trust in model predictions but also facilitates clinical interpretation, enabling researchers and clinicians to better understand the biological relevance of the extracted features and to guide decision-making in patient care.

### 4.3. The Added Value of Clinical and Demographic Information in Image-Based Prediction Models

The majority of studies focused on imaging data alone but three included studies [36,37,42] also developed models using clinical data or combining clinical and radiomic data for comparison. Interestingly, the best-performing models were those using combined data.

While Yu and colleagues [17] advocated the exclusive use of imaging data, suggesting that the inclusion of clinical information could introduce additional uncertainties into the predictive model, the evidence of these comparative studies [36,37,42] suggests that, in certain contexts, traditional clinical features may provide complementary or even superior diagnostic value compared to imaging-derived features alone.

### 4.4. The Impact of Scanner Manufacturer on the Robustness of Radiomic Features

A key finding of the Petrou et al. study [48] is that the exclusion of non-robust features consistently impaired model efficacy, suggesting that such features may carry significant predictive signal. Consequently, discarding them purely due to scanner dependency is not advisable. This underscores the need for harmonization methods like *ComBat* a statistical method used for data harmonization, specifically designed to correct batch effects, such as differences introduced by scanners, acquisition protocols, or different centers, while preserving the relevant biological information.

At the same time, it is important to recognize that data variability is not inherently detrimental. Heterogeneous, multicenter data are essential for training robust and generalizable models, as they expose algorithms to a broader range of real-world imaging conditions [56]. However, when technical variability overwhelms biological signal, model performance and reproducibility can be compromised. Therefore, a balance must be achieved between maintaining sufficient diversity to support model robustness and applying harmonization strategies to reduce non-biological noise.

### 4.5. Performance of Models and Aspects That Influence It

A key limitation identified in the literature is that many studies focus exclusively on imaging data. However, evidence demonstrates that integrating different data types significantly improves model performance. Three included studies [36,37,42] developed models that integrated clinical and radiomics data, and these combined models demonstrated the best overall performance. For instance, Boot et al. found that combined models were the most predictive for determining HPV status and for overall survival [36].

The choice of algorithms and dataset characteristics also impacts model performance, as well as the number of features. For small datasets, simpler models can outperform complex alternatives. Suh et al. [52] demonstrated that Logistic Regression (LR) and Random Forest (RF) performed better than XGBoost, attributing this to LR’s superiority with small samples and the high relevance of the selected features. The relationship between the number of features and performance is not linear. Altinok et al. [33] observed that SVM outperformed the Bayesian Network (BN), possibly due to the number of features used. However, it is noted that an excessive number of features can increase model complexity and reduce intelligibility.

Combining different sources of information, both in terms of modality and anatomical location, is an effective strategy. The combination of distinct imaging modalities, such as 18F-FDG PET/CT and MRI [43], generally yields superior results compared to using a single modality. Similarly, combining features extracted from the primary tumor and lymph nodes [16] proved more advantageous than using only one source.

A major limitation that hinders robust analysis and model comparison is the inconsistent reporting of essential performance metrics across studies. The absence of important performance metrics was previously highlighted by our group in past studies [57]. Future studies should adhere to AI-specific reporting guidelines such the Checklist for Artificial Intelligence in Medical Imaging (CLAIM) [58], the Transparent Reporting of a multivariable prediction model for Individual Prognosis Or Diagnosis (TRIPOD) [59,60,61], the Standards for Reporting Diagnostic Accuracy (STARD-AI) statement [62], and the Must AI Criteria-10 (MAIC-10) Checklist [63] to enhance reproducibility and transparency. This underscores the critical need for future studies to comprehensively report all relevant performance metrics to support robust and reliable meta-analyses. Future studies must comprehensively report all relevant performance metrics—including accuracy, sensitivity, specificity, precision, confusion matrix (values for TP, FP, FN, TN), F1-score, and AUROC—to enable robust and reliable meta-analyses.

### 4.6. Limitations of Included Studies That Affected the SR

The included studies presented several limitations:

(1) Small sample sizes were noted in several investigations, which reduces the power of generalization.
(2) Most datasets were obtained from a single center, potentially affecting reproducibility due to scanner- and protocol-related variations.
(3) Manual delineation of VOIs is subject to interobserver variability.
(4) The retrospective design carries a potential RoB, even though assessors were blinded to HPV status. However, we understand that a prospective image collection would not necessarily alter the analytical workflow.
(5) HPV detection in most studies relied on IHC p16 alone instead of combining it with at least one specific molecular test, a major clinical limitation affecting the “ground truth” label.
(6) The absence of external validation further limits the assessment of model generalizability.
(7) Explainability methods were applied in only a few studies, despite their importance for understanding model decision-making and identifying the features most influential in the predictions.

## 5. Conclusions

From a clinical perspective, radiomics-based ML models could serve as complementary or alternative tools to molecular HPV testing, particularly in resource-limited settings where tissue is unavailable or specialized tests like p16 IHC or HPV DNA analysis are inaccessible. This potential is supported by imaging traits common in HPV-positive oropharyngeal cancers—such as increased signal intensity, smaller and more spherical lesion volumes, and greater spatial heterogeneity—which are captured by the radiomic features underlying these models’ strong predictive performance.

Despite promising diagnostic results, these models remain far from clinical application, constrained by high methodological variability, suboptimal reference standards, and a lack of external validation. Future research must therefore prioritize methodological rigor, reproducibility, and transparent AI reporting. Crucially, multicenter and prospective studies employing standardized protocols, dual HPV testing and the integration of clinical data with radiomics are warranted to validate clinical utility. Moreover, integrating explainable AI frameworks will be essential to transform radiomic models from promising research tools into clinically reliable biomarkers for HPV-related oropharyngeal cancer. This approach not only offers a tool to evaluate HPV status independently but also holds promise for enabling personalized therapies, such as treatment de-escalation, by tailoring strategies to individual patient risk. The goal is not to replace molecular testing but to advance reliable radiomic biomarkers from research into routine clinical decision-making.

## Figures and Tables

**Table 1:** General characteristics of studies evaluating radiomics-based ML models for HPV status prediction in OPSCC cancer.

Author, Year [Ref.]	VOI	Patient *N*	HPV Status Test	Imaging Modality	Features	Feature Selection Method	Classifier	AUROC (SD) [CI] from Validation	AUROC (SD) [CI] from External Validation (Test)	Conclusions
Altinok et al. 2023 [33]	GTVp	Total: 246 (216 HPV+; 30 HPV−) Training: 172 Validation: 74	NI	CE-CT	2 radiomic: Sphericity and Max2DDiameterRow	*t*-test and MMPC	BN	0.72	NA	BN can predict HPV status, demonstrate probabilistic relationships between predictors (nodes), and allow for interpretability.
					25 radiomic	*t*-test and MMPC	SVM	0.83	NA	
Bagher-Ebadian et al. 2020 [11]	GTVp and GTVn	Total: 187 (116 HPV+; 71 HPV−) Training: 67% (125) External validation: 33% (62)	IHC p16	CE-CT	12 radiomic: GLCM (Contrast, Energy, Entrophy); Shape Features (MeanBreadth & SphericalDisproportion); DOST (Energy 1,2,3,4,6,7,8,9)	Levene, Kolmogorov–Smirnov’s tests, and Biserial correlation	GLM with seven PCs	NI	0.91 [0.905–0.916]	Intensity and frequency arrangements observed on CE-CT may be used as imaging signature for HPV status detection.
Bogowicz et al. 2017 [34]	GTVp	Total: 149 (62 HPV+; 87 HPV−) Training: 93 Validation: 56	IHC p16	CT	4 radiomic (*N* = 4): LLL standard deviation, LLL small zone high gray-level emphasis, HHL difference entropy, Coefficient of variation	MLR	LR	0.78 [0.77–0.79]	NA	Tumor heterogeneity is associated with local tumor control and HPV status. Radiomics-based model showed better performance than models incorporating clinical parameters.
Bogowicz et al. 2020 [35]	GTVp	Total: 1174 (unclear HPV status proportion) Training: unclear External validation: unclear	IHC p16	CT	981 radiomic: shape, intensity distribution, texture and wavelet transform	Hierarchical clustering and univariate logistic regression	LR	0.825 [0.71–0.94] **	NA	Radiomics-based models can be trained in the distributed fashion. This approach facilitates regular updates of radiomics signatures. The models showed good discrimination, but some cases may require recalibration.
Boot et al. 2023 [36]	GTVp	Total: 249 (91 HPV+; 158 HPV−) Training: unclear Internal validation: unclear	Specific testing (PCR) and IHC p16	MRI	NI	Firefly Algorithm	LR	0.62 (0.12)	NA	The model combining clinical data and radiomic factors outperformed the radiomics-only model. Models showed sufficient performance.
							RF	0.65 (0.11)		
					NI	LASSO	LR	0.79 (0.10)		
							RF	0.77 (0.10)		
					NI	PCA	LR	0.78 (0.09)		
							RF	0.74 (0.08)		
					NI	RFE-LR	LR	0.78 (0.10)		
							RF	0.79 (0.09)		
					NI	RFE-RF	LR	0.77 (0.10)		
							RF	0.80 (0.09)		
					NI	RFE-SVM	LR	0.69 (0.10)		
							RF	0.72 (0.10)		
					49 radiomic features: tumor sphericity, intensity variation, compactness, and tumor diameter	Univariate	LR	0.79 (0.09)		
							RF	0.79 (0.10)		
					NA	noFS	LR	0.77 (0.11)		
							RF	0.77 (0.10)		
					Clinical (T-stage, N-stage, smoking status, gender) + radiomic factor (1, 3, 5 and 6)	Redundancy filtering e dimensionality reduction via factor analysis (to select radiomic features)	LR	0.87		
Bos et al. 2021 [37]	GTVp	Total: 153 (76 HPV+; 59 HPV−) Training: 91 Internal validation: 62	IHC p16	MRI	6 clinical: age, gender, smoking status, T-classification (T1 + T2 vs. T3 + T4), N-classification (N0 vs. *N* > 0), tumor subsite	Wrapper-based feature selection using RFE	LR	0.794 [0.788–0.800]	NA	LR models can predict HPV status. Although the combined model performs better, the clinical model is easier to implement.
					14 radiomic: 77 stable, filtered radiomic features extracted from post-contrast 3DT1W MRI		LR	0.764 [0.758–0.770]	NA	
					Combined		LR	0.871 [0.866–0.876]	NA	
Bos et al. 2022 [38]	GTVp	Total: 153 (76 HPV+; 77 HPV−) Training: 91 Internal validation: 62	IHC p16, p53	MRI	1184 radiomic features initially; 4 to 77 features, depending on the delineation strategy.	RFE	LR	0.84 [0.83–0.85]	NA	Alternative delineations can substitute labor and time intensive full tumor delineations. Models perform better in large delineations. The loss of shape and size features might be adequately compensated with textural features.
Fanizzi et al. 2024 [39]	GTVp	Training: 499 (356 HPV+; 143 HPV−) Independent test (external validation): 92 (43 HPV+; 49 HPV−)	IHC p16	CT	NA	NA	Inception-v3 ^MA^	NI	0.735	Most important areas for HPV status prediction are inside the GTV and at the edges. XAI contributes to increasing confidence in using computer-based predictive models in clinical practice.
Haider et al. 2020 [40]	GTVp	Total: 435 Training: 326 (244 HPV+; 82 HPV−) Validation: 80 (60 HPV+; 20 HPV−) External validation: 29 (11 HPV+; 18 HPV−)	Specific testing (DNA ISH or PCR) and/or IHC p16	PET/CT	NI	RIDGE	XGBoost	0.77 [0.65–0.89]	NI	PET-based radiomics signatures yielded similar classification performance to CT-based models, with potential added value from combining PET- and CT-based radiomics for prediction of HPV status.
				PET	NI	RIDGE	Naive Bayes	0.70 [0.55–0.85]	0.75 [0.55–0.94]	
				CT	NI	RIDGE	XGBoost	0.76 [0.63–0.88]	NI	
	GTVn	Total: 741 metastatic lymph nodes Training: 518 Validation: 148 External validation: 75		PET/CT	NI	Hierarchical clustering	ElNet	0.61 [0.49–0.73]	NI	
				PET	NI	Hierarchical clustering	XGBoost	0.60 [0.50–0.71]	0.58 [0.45–0.71]	
				CT	NI	Hierarchical clustering	ElNet	0.59 [0.48–0.70]	NI	
	GTVp + GTVn			PET/CT	NI	noFS	XGBoost	0.75 [0.62–0.87]	NI	
				PET	NI	noFS	XGBoost ^MA^	0.71 [0.58–0.85]	0.83 [0.68–0.98]	
				CT	NI	noFS	XGBoost	0.67 [0.54–0.80]	NI	
	GTVn (all lymph nodes)			PET/CT	NI	pMIM	XGBoost	0.60 [0.42–0.78]	NI	
				PET	NI	Hierarchical clustering	XGBoost	0.65 [0.47–0.84]	0.73 [0.47–0.94]	
				CT	NI	pMIM	XGBoost	0.61 [0.44–0.77]		
Haider et al. 2024 [41]	GTVp	Total: 430 Training: 325 Validation: 79 External validation: 26	Specific testing (DNA ISH or PCR) and/or IHC p16	^18^F-FDG PET	Radiomic (*N* = x): shapes (*N* = 14), 198 first-order features (*N* = 198), and texture features (*N* = 825) per patient per PET intensity normalization technique.	MRMR	XGBoost ^MA^	0.72 [0.58–0.87]	0.78 [0.60–0.96]	Normalization technique may affect the radiomic features’ predictive value. A few features were reproducible across intensity normalization techniques (uniform normalization is a prerequisite).
Huang et al. 2019 [42]	GTVp	Total: 166 Training and validation: 113 External validation: 53	VirusSeq for the TCGA-HNSCC cohort; IHC p16 for The Stanford-HNSCC dataset	CT	Radiomic features: 540 total, 491 retained after robustness testing) from 5 groups (shape/size, first-order, histogram, texture, wavelet).	Intraclass correlation coefficient with the 95%CI > 0.9, the minimum redundancy maximum relevance, and LASSO	LR ^MA^	NI	0.76 [0.60–0.91]	Quantitative image features are capable of distinguishing several molecular phenotypes. Radiomics can potentially serve as a non-invasive tool to identify treatment-relevant subtypes.
					Clinical features: age, gender, smoking status, primary tumor site, TNM staging.			NI	0.86 [0.74–0.98]	
					Radiomic + clinical			NI	0.88 [0.78–0.98]	
Jo et al. 2023 [43]	GTVp	Total: 126 (103 HPV+; 23 HPV−) Training: 75 Internal validation: 51	IHC p16	MRI	Radiomic (*N* = 293): post-contrast 3D T1WI (*N* = 93); T2WI (*N* = 93); shape (*N* = 14)	NI	LightGBM	0.762 [0.564–0.959]	NI	The most relevant variable associated with HPV-negative status was SUVmax. 18F-FDG PET/CT exhibited incremental value in predicting HPV status when combined with an MRI radiomics model (multimodal approaches performs better).
				^18^F-FDG PET/CT	Metabolic and volume-based parameters: SUVmax, SUVmean, SUVpeak, MTV, TLG. Heterogeneity parameters: SD, skewness, kurtosis, heterogeneity index, coefficient of variation.		LightGBM	0.638 [0.404–0.871]	NA	
				MRI and ^18^F-FDG PET/CT	A combination of MRI radiomic features and ^18^F-FDG PET/CT parameters		LightGBM	0.823 [0.668–0.978]	NA	
Lang et al. 2021 [44]	GTVp	Total: 850 (607 HPV+; 238 HPV−) Training: 675 (513 HPV+; 162 HPV−) Validation: 90 (71 HPV+; 19 HPV−) External validation: 80 (23 HPV+; 57 HPV−)	Specific testing (DNA ISH) and/or IHC p16	CT	NA	NA	3D CNN pre-trained with sports video clips ^MA^	NI	0.81 (0.02) [0.77–0; 84]	Improved performance of the 3D approach over the 2D approach (the third dimension contains essential information)
						NA	3D CNN (no pre-training)	NI	0.64 (0.05)	
						NA	2D CNN (VGG16) pre-trained with Imagenet	NI	0.73 (0.02)	
Leijenaar et al. 2018 [45]	GTVp	Total: 778 (426 HPV+; 352 HPV−) Training: 628 (344 HPV+; 284 HPV−) Internal validation: 150 (82 HPV+; 68 HPV−)	IHC p16	CE-CT	- 902 radiomic features: tumor intensity, shape, texture, wavelet, Laplacian of Gaussian. - 165 features were used in the model trained with all data (including patients with artifacts)—referred to as the M_all_ model. - 173 features were used in the model trained with data excluding artifacts—referred to as the M_no art_ model. - Feature reduction: Highly correlated features (ρ > 0.9) removed. Final model used 37 and 50 features in Mall and Mno_art, respectively.	Pearson correlation to remove the variables with the largest mean absolute correlation and LASSO	LR	Trained in M_all,_ Validated in M_no art_ subset: 0.7658 [0.6592–0.8724]	NA	Molecular information can be derived from standard medical images and shows potential for radiomics as imaging biomarker of HPV status.
							LR	Trained in M_no art,_ Validated in M_no art_ subset: 0.8005 [0.6967–0.9044]	NA	
Li et al. 2023 [16]	GTVp and GTVn	Total: 141 (78 HPV+; 63 HPV−) Training: 116 (62 HPV+; 54 HPV−) External validation: 25 (16 HPV+; 9 HPV−)	IHC p16	MRI (CE-T1WI-based)	2092 radiomics features: original, Laplacian of Gaussian, wavelet, shape, histogram (first-order statistical), and texture features (derived from GLCM, GLRLM, GLDM, GLSZM)	RFE	SVM	NI	0.80 (0.10) [0.55–0.94]	Fusion models (features extracted from both primary tumors and lymph nodes) and models based on multisequence imaging yielded better performance.
				Mari (T2WI-based)		RFE	SVM	NI	0.74 (0.10) [0.51–0.95]	
				MRI (Multisequence-based)		RFE	SVM ^MA^	NI	0.91 (0.10) [0.72–0.98]	
Lv et al. 2022 [46]	GTVp and GTVn	Total: 160 Training: 100 (72 HPV+; 28 HPV−) Testing (not external validation): 60 (38 HPV+; 22 HPV−)	IHC p16	^18^F-FDG PET/CT	497 radiomics features: shape, histogram-base, and texture features	ranking features by descending AUC, selecting the top 2–20 non-redundant features, and choosing the final multivariate logistic regression model based on the highest AUC and lowest Bayesian Information Criterion	Multivariate LR	NI	Original image 0.484	Saliency-guided PET/CT radiomics are feasible to predict outcome confirming that certain regions are more relevant to tumor aggressiveness and prognosis. The radiomics score as a surrogate of HPV status also conveyed prognostic information.
							Multivariate LR	NI	Fused image 0.653	
Park et al. 2022 [47]	GTVp	Total: 155 (136 HPV+; 19 HPV−) Training: 108 (95 HPV+; 13 HPV−) Internal validation: 47 (41 HPV+; 6 HPV−)	IHC p16	MRI	8 radiomics features	RFE	LightGBM	0.8333	NA	Satisfactory performance in predicting the HPV status. Absence of external validation is a limitation.
					23 radiomics features	RFE	LR	0.792	NA	
Petrou et al. 2024 [48]	Cubic ROI of the oropharynx, not precise anatomical delineation	Total: 50 Training: 40 (23 HPV+; 17 HPV−) Validation: 10 (5 HPV+; 5 HPV−)	NI	CT	NA	NA	ResNet-18	NI	NA	CT scans analyzed by DL models hold promise for non-invasive HPV detection in HNC.
					First-order features (Entropy, Kurtosis, Mean, Skewness, Uniformity). Second-order features related to the Gray-Level Co-occurrence Matrix (Contrast, Correlation, Inverse Variance, Joint Energy), the Gray-Level Size Zone Matrix (Small Area Emphasis, Large Area Low Gray-Level Emphasis), and the Neighboring Gray Tone Difference Matrix (Busyness, Complexity, Coarseness, Contrast).	NA (the authors preselected 15 radiomic features based on prior literature, without performing any statistical or algorithmic selection procedures)	K-Nearest Neighbors	NI	NA	
							LR	NI	NA	
							Decision tree	NI	NA	
							RF	NI	NA	
Prasse et al. 2023 [18]	GTVp, GTVn and parotid.	Total: 53 (20 HPV+; 33 HPV−) Training: 40 Internal validation: 13 cases	IHC p16	CT	First order, shape and texture (GLCM)	Shapley values	MLP	GTVp: 0.71	NI	Classification performances of HPV status based on non-contrast CT imaging of the parotid gland and TM and LNM are comparable, suggesting involvement of the parotid in HPV infections of the oropharyngeal region. Generalization gap.
							RF	Parotid: 0.76	NI	
							XGBoost	GTVn: 0.82	NI	
							XGBoost	All: 0.86	NI	
Reiazi et al. 2021 [49]	GTVp	Total: 1294 (824 HPV+; 470 HPV−) Training: 776 Internal validation: 259 Additional validation (not external): 259	IHC p16	CT	1874 radiomic features: 14 shape, 360 first order, 480 GLCM, 320 GLRLM + GLSZM, 280 GLDM, 100 NGTDM. After selection, GLDM e NGTDM were the most relevant.	mRMR Ensemble and Wilcoxon test with FDR correction (Bonferroni)	RF	Toshiba–Mix: 0.79	NI	Radiomic features are significantly affected by scanner type, even when acquisition parameters are identical. t-SNE clustering and Wilcoxon tests showed clear separation of features by scanner manufacturer, indicating domain dependency. The best performance occurred when the training set was scanner-specific (Toshiba)
								GE-Mix: 0.75	NI	
								Mix-Toshiba 0.78	NI	
								Mix-GE: 0.76	NI	
								GE-GE:0.74	NI	
								Toshiba-Toshiba: 0.75	NI	
								GE-Toshiba: 0.73	NI	
								Toshiba–GE: 0.70	NI	
								Mix–Mix (with robust features): 0.73	NI	
								RF (Mix training and validation): 0.74	NI	
Sim et al. 2023 [50]	GTVp	Total: 154 (133 HPV+; 21 HPV−) Training: 110 External validation: 44	IHC p16	MRI (ADC-histogram)	293 radiomic features: 14 shape, 18 first order, 24 GLCM, 14 GLDM, 16 GLSZM, 16 GLRLM, 5 NGTDM.	No explicit feature selection algorithm specified.	Ridge	NI	0.791 [0.775–0.808]	The ADC radiomics model has a potential for generalizability and applicability in clinical practice. Exploring multiple oversampling and ML techniques was a valuable strategy for optimizing radiomics model performance.
				MRI (CE-T1WI)			RF	NI	0.604 [0.590–0.618]	
				MRI (T2WI)			Fully connected network ^MA^	NI	0.695 [0.673–0.717]	
				MRI (CE-T1WI + T2WI)			RF ^MA^	NI	0.642 [0.626–0.657]	
				MRI (ADC-histogram)			Ridge—Conventional histogram model	NI	0.734 [0.707–0.760]	
Sohn et al. 2021 [51]	GTVp	Total: 62 (52 HPV+; 10 HPV−) Training: 43 Internal validation: 19	IHC p16	MRI	First-order skewness, GLCM (informational measure of correlation 1), NGTDM (coarseness), shape flatness from post-contrast 3D T1WIs, and first-order skewness and NGTDM strength from T2WIs	LASSO penalization	LR	0.744 [0.496–0.991]	NA	The model can potentially aid in treatment planning without the need for invasive procedures.
Suh et al. 2020 [52]	GTVp and GTVn	Total: 60 (48 HPV+; 12 HPV−) Training: 40 (32 HPV+; 8 HPV−) Internal validation: 20 (16 HPV+; 4 HPV−)	Specific testing (PCR) and/or IHC p16	MRI	Entropy_std, Autocorrelation_std, Correlation_std, Homogeneity1_std, Entropy_std, Correlation, Difference variance	LASSO penalization	LR	0.77 [0.50, 0.96]	NA	ADC-histogram analysis can differentiate tumor heterogeneity in HPV+ vs. HPV-tumors. MRI-based radiomic signatures can serve as non-invasive biomarkers for HPV status.
						LASSO penalization	RF	0.76 [0.47, 0.97]	NA	
						LASSO penalization	XGBoost	0.71 [0.50, 0.93]	NA	
Yu et al. 2017 * [17]	GTVp and GTVn	Total: 315 Training: 150 (128 HPV+; 22 HPV−) Internal validation: ≈82 ** Final validation (not external): ≈83 **	IHC p16	CT	MeanBreadth and SphericalDisproportion	LR	Wilcoxon and Kolmogorov–Smirnov tests, biserial correlation, marginal AUC ranking, and forward selection ^MA^	NI	0.91549	HPV+ patients have smaller and geometrically simpler tumor. The authors developed a framework based on statistical radiomics approach to predict HPV status, winning the MICCAI grand challenge.

** Calculated by the authors based on data available in the article. * MICCAI grand challenge: Medical Image Computing and Computer Assisted Intervention; ^MA^: selected for meta-analysis; ^18^F-FDG PET: ^18^F-fluorodeoxyglucose positron emission tomography; 3DT1W MRI: three-dimensional T1-weighted Magnetic Resonance Imaging; AUROC: area under the receiver operating characteristic curve; AUC: area under the curve; BN: Bayesian Network; CE-CT: contrast-enhanced computed tomography; CI: (95% CI); CT: computed tomography; ElNet: Logistic regression with elastic net regularization; GLCM: Gray-Level Co-occurrence Matrix; GLDM: Gray-Level Dependence Matrix; GLM: generalized linear model; GLRLM: Gray-Level Run Length Matrix; GLSZM: Gray-Level Size Zone Matrix; GTVn: gross nodal tumor volume; GTVp: gross primary tumor volume; HHL difference entropy: Difference entropy from the HHL (High-High-Low) wavelet-transformed image; CE-T1WI: contrast-enhanced T1-weighted images; HNSCC: head and neck squamous cell carcinoma; IHC: immunohistochemistry; ISH: in situ hybridization; KNN: K-Nearest Neighbors; LASSO: Linear regression with the least absolute shrinkage and selection operator; LLL small zone high gray-level emphasis: Small zone high gray-level emphasis from the LLL wavelet-transformed image; LLL standard deviation: Standard deviation of the LLL (Low-Low-Low) wavelet-transformed image; LR: Logistic Regression; MLP: multilayer perceptron; MLR: multivariable logistic regression; MMPC: Max-Min Parents and Children; MRI: Magnetic Resonance Imaging; MRMR: minimum-redundancy-maximum-relevance; MRMRe: Parallelized Minimum Redundancy, Maximum Relevance; MTV: Metabolic Tumor Volume (i.e., the sum of voxels above 40% of SUVmax); NA: not applicable; NGTDM: neighborhood gray tone difference matrix; NGTDM: neighboring gray-tone difference matrix; NI: not informed; noFS: no feature selection applied; PCA: principal component analysis; PCR: polymerase Chain Reaction; PCs: principal components; PET/CT: Positron Emission Tomography combined with Computed Tomography (PET/CT); pMIM: Pearson correlation-based redundancy reduction combined with a mutual information maximization filter; Pre: precision; RF: random forest; RFE: recursive feature elimination; RIDGE: Logistic regression with RIDGE regularization adapted for feature selection; ROI: region of interest; SD: standard deviation; SD: standard deviation; Sen: sensitivity; Spe: specificity; SUVmax: maximum standardized uptake value (i.e., the most intense metabolic area); SUVmean: average SUV within the VOI; SUVpeak: average SUV within a small fixed-size region of highest uptake; SVM: support vector machine; T2WI: T2-weighted images; TLG: Total Lesion Glycolysis (i.e., SUVmean × number of voxels); VOI: volume of interest; XGBoost: eXtreme Gradient Boosting.

**Table 2:** Risk of bias assessment of the studies included in the SR.

	ROB				Applicability				
Author/Year [Ref.]	Participants	Predictors	Outcomes	Analysis	Participants	Predictors	Outcomes	ROB	Applicability
Altinok et al. 2023 [33]	+	?	−	−	+	+	+	−	+
Bagher-Ebadian et al. 2020 [11]	+	+	−	−	+	+	+	−	+
Bogowicz et al. 2017 [34]	+	+	−	−	+	+	+	−	+
Bogowicz et al. 2020 [35]	+	+	−	−	+	+	+	−	+
Boot et al. 2023 [36]	+	+	+	−	+	+	+	−	+
Bos et al. 2021 [37]	+	?	?	−	+	+	+	−	+
Bos et al. 2022 [38]	+	+	−	?	+	+	+	−	+
Fanizzi et al. 2024 [39]	+	?	−	?	+	+	+	−	+
Haider et al. 2020 [40]	+	+	?	?	+	+	+	?	+
Haider et al. 2024 [41]	+	+	?	?	+	+	+	?	+
Huang et al. 2019 [42]	+	+	−	+	+	+	+	−	+
Jo et al. 2023 [43]	+	+	−	+	+	+	+	−	+
Lang et al. 2021 [44]	+	?	−	?	+	+	+	−	+
Leijenaar et al. 2018 [45]	+	?	−	+	+	+	+	−	+
Li et al. 2023 [16]	+	+	−	−	+	+	+	−	+
Lv et al. 2022 [46]	+	?	−	?	+	+	+	−	+
Park et al. 2022 [47]	+	?	−	?	+	+	+	−	+
Petrou et al. 2024 [48]	?	?	?	−	+	+	+	−	+
Prasse et al. 2023 [18]	+	+	−	−	+	+	+	−	+
Reiazi et al. 2021 [49]	+	+	−	?	+	+	+	−	+
Sim et al. 2023 [50]	+	+	−	?	+	+	−	−	+
Sohn et al. 2021 [51]	+	?	−	+	+	+	+	−	+
Suh et al. 2020 [52]	+	?	?	+	+	+	+	?	+
Yu et al. 2017 [17]	+	+	−	−	+	+	+	−	+

PROBAST = Prediction model Risk Of Bias ASsessment Tool; ROB = risk of bias. * + indicates low ROB/low concern regarding applicability; − indicates high ROB/high concern regarding applicability; and ? indicates unclear ROB/unclear concern regarding applicability.

**Table 3:** Summary of true positive (TP), false negative (FN), false positive (FP), and true negative (TN) counts extracted from the selected studies that reported external validation results and were used to calculate the pooled performance metrics in the meta-analysis.

Model Category	Architecture	Author	TP	FN	FP	TN
DL models	Inception-v3	Fanizzi et al. 2024 [39]	28	32	17	15
	3D CNN (pre-trained)	Lang et al. 2021 [44]	24	8	13	35
	Fully connected network	Sim et al. 2023 [50]	15	12	1	16
Traditional models	GLM with seven PCs	Bagher-Ebadian et al. 2020 [11]	28	5	5	24
	LR	Huang et al. 2019 [42]	13	3	10	27
	SVM	Li et al. 2023 [16]	12	4	1	8

GLM: generalized linear model; LR: Logistic Regression; PCs: principal components; SVM: Support Vector Machine. Observation: When not explicitly provided, these values were calculated by the authors based on the available performance data.

## Data Availability

No new data were created or analyzed in this study. Data sharing is not applicable to this article.

## Supplementary Materials

The following supporting information can be downloaded at: https://www.mdpi.com/article/10.3390/diagnostics16010068/s1, Table S1: PRISMA 2020 Checklist for the Systematic Review.

## Abbreviations

ADC	Apparent Diffusion Coefficient
AI	Artificial Intelligence
AJCC	American Joint Committee on Cancer
ASCO	American Society of Clinical Oncology
AUROC	Area Under the Receiver Operating Characteristic Curve
BN	Bayesian Network
BVS	Biblioteca Virtual em Saúde
CE-CT	Contrast-Enhanced Computed Tomography
CI	Confidence Interval
CLAIM	Checklist for Artificial Intelligence in Medical Imaging
CNN	Convolutional Neural Network
CRD	Centre for Reviews and Dissemination
CT	Computed Tomography
CUP	Cancer of Unknown Primary
DL	Deep Learning
DNA-ISH	DNA In Situ Hybridization
DWI	Diffusion-Weighted Imaging
F1-score	Harmonic Mean of Precision and Recall
FDG	Fluorodeoxyglucose
FN	False Negative
FP	False Positive
GLCM	Gray-Level Co-occurrence Matrix
GLSZM	Gray-Level Size Zone Matrix
GTVn	Gross Nodal Tumor Volume
GTVp	Gross Primary Tumor Volume
HPV	Human Papillomavirus
IHC	Immunohistochemistry
LASSO	Least Absolute Shrinkage and Selection Operator
LoG	Laplacian of Gaussian
LR	Logistic Regression
MAIC-10	Must AI Criteria-10
ML	Machine Learning
MRI	Magnetic Resonance Imaging
OPSCC	Oropharyngeal Squamous Cell Carcinoma
p16	Cyclin-Dependent Kinase Inhibitor 2A
PCR	Polymerase Chain Reaction
PET	Positron Emission Tomography
PET/CT	Positron Emission Tomography–Computed Tomography
PICOS	Population, Intervention, Comparator, Outcome, Study Design
PRISMA	Preferred Reporting Items for Systematic Reviews and Meta-Analyses
PRISMA-DTA	Preferred Reporting Items for Systematic Reviews and Meta-Analyses of Diagnostic Test Accuracy
PRISMA-P	Preferred Reporting Items for Systematic Review and Meta-Analysis Protocols
PROBAST	Prediction Model Risk of Bias Assessment Tool
PROSPERO	International Prospective Register of Systematic Reviews
QCRI	Qatar Computing Research Institute
RF	Random Forest
RFE	Recursive Feature Elimination
RoB	Risk of Bias
ROI	Region of Interest
SHAP	Shapley Additive exPlanations
SMOTE	Synthetic Minority Over-sampling Technique
SR	Systematic Review
sROC	Summary Receiver Operating Characteristic
STARD-AI	Standards for Reporting Diagnostic Accuracy for Artificial Intelligence
SUVmax	Maximum Standardized Uptake Value
SVM	Support Vector Machine
t-SNE	t-distributed Stochastic Neighbor Embedding
TN	True Negative
TNM	Tumor–Node–Metastasis
TP	True Positive
TRIPOD	Transparent Reporting of a multivariable prediction model for Individual Prognosis Or Diagnosis
UICC	Union for International Cancer Control
VOI	Volume of Interest
XAI	Explainable Artificial Intelligence
XGBoost	eXtreme Gradient Boosting

## Appendix A. General Search Keywords

**Table A1:** PICO strategy.

Acronym PICO	Keywords
Patients	“oral cancer” OR “oropharyngeal cancer”
Intervention/Exposition	radiomics AND “machine learning” OR “artificial intelligence” OR “deep learning” OR “convolutional neural network” OR “artificial neural network”
Comparison	Not applicable
Outcome	HPV OR “HPV status”

After multiple attempts to reorganize the search keywords, we opted for the combination of the terms “radiomic” and “HPV,” which yielded the most comprehensive results across the databases, thereby ensuring broader coverage of the research topic.

**Table A2:** Search strategy.

Database	Query Search Date: 7 November 2024	Retrieved Studies (n)
Cochrane	radiomics AND HPV	7
Embase	(‘radiomics’/exp OR radiomics) AND hpv	155
IEEE Xplore	(“Full Text & Metadata”:radiomics AND “Full Text & Metadata”:human papillomavirus OR HPV)	205
Portal Regional da Biblioteca Virtual em Saúde	radiomics OR radiômica OR radiomica AND HPV OR “human papillomavirus” OR “papilomavírus humano” OR “ virus del papiloma humano”	15
PubMed	(radiomics) AND (HPV)	87
Scopus	(radiomics) AND (HPV)	92
Web of Science	radiomics (Topic) and HPV (Topic)	103
arXiw	order: -announced_date_first; size: 50; classification: Computer Science (cs); include_cross_list: True; terms: AND title=RADIOMICS; AND title=human papillomavirus; OR title=HPV	7
Google Scholar	radiomics AND HPV	99
ProQuest	title(radiomics) AND title(HPV)	7
**TOTAL**		**777**

## Appendix B. Excluded Articles and Reasons for Exclusion

**Table A3:** Excluded articles and reasons for exclusion.

	Author	Title	Year	Country	Journal/Conference Name	Exclusion Criteria
1	Bologna et al.	Prognostic radiomic signature for head and neck cancer: Development and validation on a multi-centric MRI dataset	2023	Italy	Radiotherapy and Oncology.	Other outcome prediction (not HPV status)
2	Giannitto et al.	Association of quantitative MRI-based radiomic features with prognostic factors and recurrence rate in oropharyngeal squamous cell carcinoma	2020	Italy	Neoplasma	Only correlated radiomic features with prognostic factors
3	Leijenaar et al.	Radiomics in OPSCC: a novel quantitative imaging biomarker for HPV status?	2016	Italy	ESTRO 35 2016	Conference Abstract
4	Parmar et al.	Radiomic feature clusters and Prognostic Signatures specific for Lung and Head & Neck cancer	2015	USA	Scientific Reports	Only identification of radiomic signatures
5	Buch et al.	Using Texture Analysis to Determine Human Papillomavirus Status of Oropharyngeal Squamous Cell Carcinomas on CT	2014	USA	Head and Neck	Conventional statistical analysis rather than machine learning
6	Ou et al.	Predictive and prognostic value of CT based radiomics signature in locally advanced head and neck cancers patients treated with concurrent chemoradiotherapy or bioradiotherapy and its added value to Human Papillomavirus status	2017	France	Oral Oncology	Other outcome prediction (not HPV status)
